# First ever case report of co-occurrence of hobnail variant of papillary thyroid carcinoma and intrathyroid parathyroid adenoma in the same thyroid lobe

**DOI:** 10.1016/j.ijscr.2020.04.025

**Published:** 2020-05-05

**Authors:** Omer Al-Yahri, Abdelrahman Abdelaal, Walid El Ansari, Hanan Farghaly, Khaled Murshed, Mahmoud A. Zirie, Mohamed S. Al Hassan

**Affiliations:** a Department of General Surgery, Hamad General Hospital, Doha, Qatar; b Department of Surgery, Hamad General Hospital, Doha, Qatar; c College of Medicine, Qatar University, Doha, Qatar; d School of Health and Education, University of Skövde, Skövde, Sweden; e Department of Lab Medicine & Pathology, Hamad General Hospital, Doha, Qatar; f Department of Endocrinology, Hamad General Hospital, Doha, Qatar

**Keywords:** Hobnail papillary thyroid carcinoma, Parathyroid adenoma, Neck mass, Molecular profile, BRAF^V600E^ mutation

## Abstract

**Introduction:**

The hobnail variant of papillary thyroid cancer (PTC) is rare. Intrathyroid parathyroid adenoma (ITPA) is also rare. Co-ocurrence of PTC and ITPA in the same thyroid lobe is extremely rare. Likewise, primary hyperparathyroidism with such non-medullary thyroid carcinoma is rare. The specific molecular profile of hobnail PTC (HPTC) is different from the classic, poorly differentiated and anaplastic variants and may contribute to its aggressive behavior. HPTC’s genetic profile remains unclear.

**Presentation of case:**

A 61-year-old woman presented to our endocrine clinic with generalized aches, bone pain, polyuria, and right neck swelling of a few months’ duration. Laboratory findings revealed hypercalcemia and hyperparathyroidism. Ultrasound of the neck showed 4.6 cm complex nodule within the right thyroid lobe. Sestamibi scan suggested parathyroid adenoma in the right thyroid lobe. Fine-needle aspiration (FNA) revealed atypical follicular lesion of undetermined significance. She underwent right lobectomy, which normalized the intraoperative intact parathyroid hormone levels. Final pathology with immunohistochemical stains demonstrated HPTC and IPTA (2 cm each). Next-generation sequencing investigated the mutation spectrum of HPTC and detected BRAFV600E mutation.

**Conclusions:**

A parathyroid adenoma should not exclude the diagnosis of thyroid carcinoma. Thyroid evaluation is needed for patients with primary hyperparathyroidism to prevent missing concurrent thyroid cancers. Cytomorphologic features to distinguish thyroid from parathyroid cells on FNA cytology must be considered. Immunohistochemical stains are important. BRAFV600E is the most common mutation in HPTC. This is possibly the first reported case of HPTC and ITPA co-occurring within the same thyroid lobe. Studies that define other molecular abnormalities may be useful as therapeutic targets.

## Highlights

• First reported case of hobnail variant of papillary thyroid cancer and Intrathyroid parathyroid adenoma occurring within same thyroid lobe.
• Next-generation sequencing of the mutation spectrum of hobnail variant of papillary thyroid cancer showed BRAFV600E mutation.
• Studies that define other molecular abnormalities may be useful as therapeutic targets.

## 1 Introduction

Papillary thyroid cancer (PTC) is the most common type of endocrine malignancy. Its incidence ranges from 0.3% to 2.7%, and mean age is 57 years (range 17–87 years) [1]. Its prognosis is usually favorable [[2], [3], [4], [5], [6]]. The vast majority of PTC variants are well-differentiated (WD), such as papillary and follicular variants, with survival rates of approximately 95% at 40 years [7]. Despite that, 15–20% of WD variants become radioiodine refractory (RAI-R) and no other therapeutic options are available at this time [8].

The hobnail variant of papillary thyroid carcinoma (HPTC) is a rare entity [[9], [10], [11], [12], [13], [14], [15], [16], [17], [18]], has a prevalence of < 2%, and is usually more aggressive than classical PTC [12,19,20]. Compared to the WD PTC, HPTC displays aggressive clinical behavior in the form of large tumor size, lymph node metastasis, local recurrence, distant metastasis, radioactive iodine refractoriness, disease progression, worse outcome and higher mortality [10,16,17,19,20].

HPTC usually presents with neck swelling, pain, compressive symptoms, or cervical lymphadenopathy. Presentations such as tracheal invasion, acute massive hemoptysis, and intratracheal thrombosis are also encountered [15,21]. HPTC histological hallmark is a predominance of cells with hobnail appearance arranged in a micropapillary pattern [16]. Micropapillae are lined by elongated cells with high nuclear/cytoplasmic ratio and apically placed nuclei that produce a hobnail appearance [10]. Hobnail pattern with micropapillary structures was described as a loss of cellular polarity/cohesiveness [22]. Diagnosis of HPTC requires ≥ 30% hobnail-micropapillary pattern in the tumor, although minor hobnail micropapillary features (5–30%) are significant. PTC with hobnail feature ≤ 30% is less aggressive than HPTC with ≥ 30% hobnail feature but still has a poor prognosis [10,19].

Intrathyroid parathyroid adenoma (ITPA) is rare, situated totally within the thyroid, surrounded by thyroid parenchyma, with an incidence of < 1% of all hyperparathyroidism cases [23,24]. Some authors categorize ITPA into complete type, completely wrapped by thyroid tissue; and incomplete type, wrapped in ≥ 50% by thyroid tissue. Parathyroid adenoma (PA) that are wrapped in ≤ 50%, lie under the pseudocapsule or sheath covering the thyroid gland are not considered ITPA [25,26]. ITPA usually results in primary hyperparathyroidism (PHPT) and presents with asymptomatic hypercalcemia on routine screening. However, atypical presentations include calcium homeostasis disturbances or normocalcemic PHPT. The classical manifestations of PHPT (bones, stones, abdominal moans, psychic groans) are frequently encountered in developing countries, and hypercalcemic symptoms could be present e.g. anorexia, nausea, constipation, polydipsia, and polyuria [[27], [28], [29], [30]].

HPTC cases are documented [4,[9], [10], [11], [12], [13], [14], [15], [16], [17], [18]], and many ITPA cases have been published. However, only 2 cases have been reported as a simultaneous co-occurrence of PTC and ITPA [31,32]. To the best of our knowledge, there are no published reports of the simultaneous coexistence of HPTC and ITPA in the same thyroid lobe. This paper reports the first case of co-occurrence of HPTC and ITPA in the same thyroid lobe and further defines the clinical/molecular characteristics of HPTC that may be useful for prognostic stratification and may provide therapeutic targets. We report this case in line with the updated consensus-based surgical case report (SCARE) guidelines [33].

## 2 Patient presentation

A 61-year-old woman presented to the endocrinology clinic with generalized bone ache, polyuria and right neck mass for the last few months. There was no past history of kidney stones and she did not report other symptoms. She had a history of hypertension and dyslipidemia on treatment; otherwise, there were no contributory chronic medical diseases or past surgical intervention. There was no family history of thyroid cancer. On physical examination, she was vitally stable, good body built, very good physical performance, and neurological examination was unremarkable. She had a smooth, non-tender right neck swelling (3–4 cm) that moved with swallowing.

## 3 Investigations

### 3.1 Blood

Revealed high corrected calcium (2.74 mmol/L), high intact PTH (111 pg/mL). Vitamin D was 44 ng/mL, and TSH and T4 were both normal. CBC, liver and kidney functions were unremarkable.

### 3.2 Ultrasound of the neck

Large complex nodule in right thyroid lobe (4.6 × 2.4 cm) with cystic component, focal dense area of calcification, and peripheral vascularity. Left thyroid lobe showed few small hypoechoic nodules, the largest was 3.5 mm. Isthmus was unremarkable. There were small non-significant lymph nodes along left upper jugular and both upper cervical regions. No mass was observed in bilateral parathyroid regions.

### 3.3 Parathyroid sestamibi scan

**Fig. 1:**
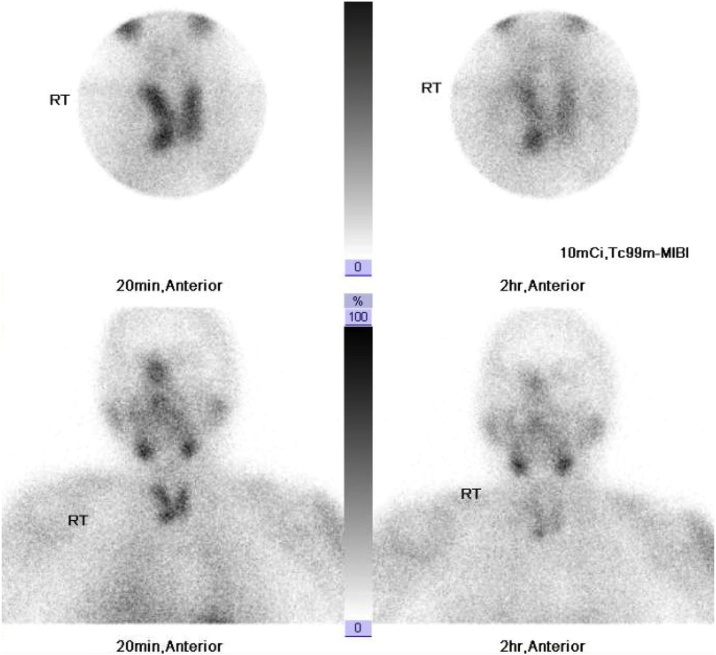
Early and late 99mTc MIBI Parathyroid Scan images of neck and mediastinum anteriorly at 20 min and 2 h.

Early and late anterior images of neck and mediastinum taken at 20 min and 2 h after IV injection of 10 mCi radiotracer. Normal early thyroid tissue uptake and late physiological washout were observed with features of high uptake and retained focal activity related to the lower pole of the right thyroid lobe, suggestive of right inferior PA. There was also a big cold nodule projecting from the lateral border of the right thyroid lobe, suggestive of thyroid nodule (Fig. 1).

### 3.4 Cytopathology

Ultrasound-guided fine-needle aspiration of the complex nodule in right lobe was done. About 10 mL of straw-colored fluid was aspirated from the cystic part. The calcified solid part was also aspirated. The sample was processed as 4 fixed slides (pap stain) and 4 air-dried slides (DiffQuik). Microscopically, there were some Hurthle cells clusters with abnormal features, few follicular cells, macrophages, minimal colloid, and blood, consistent with atypical follicular lesion of undetermined significance (FLUS).

## 4 Surgical technique

The case was discussed at the thyroid multi-disciplinary meeting and it was decided to conduct right hemithyroidectomy with removal of the right inferior PA. Under general anesthesia, conventional neck exploration did not reveal the PA, and surgery proceeded with right hemithyroidectomy as planned, which was followed by a sudden drop of intraoperative rapid PTH (94.3% drop from pre-incision serum baseline, confirming excision of ITPA). Surgery was concluded. The patient recovered smoothly with no peri-operation complications. An experienced senior surgeon undertook the procedure.

## 5 Pathology

### 5.1 Final histopathology of right thyroid lobe

**Fig. 2:**
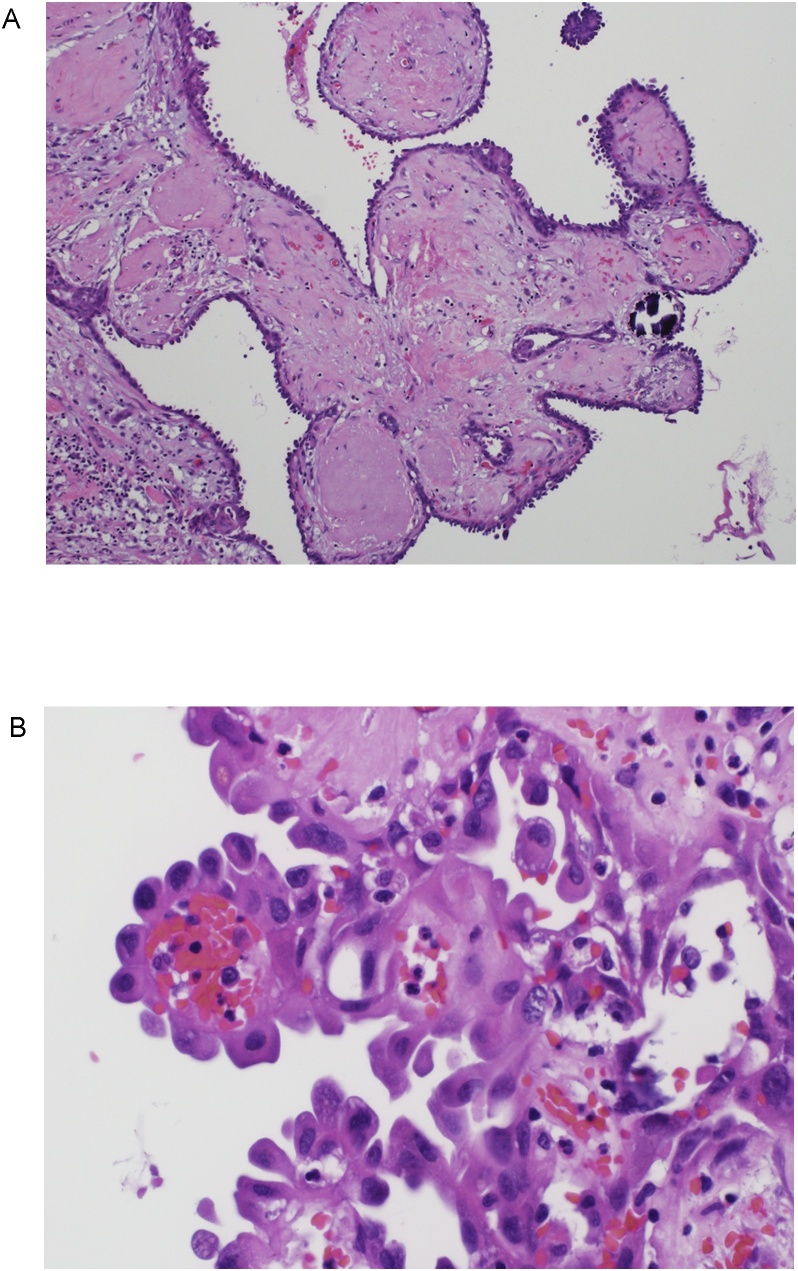
A. photomicrograph depicting papillary structures with hyalinized stalks lined by epithelial cells exhibiting hobnailing into cystic spaces. A psammomatous calcification is also present (hematoxylin and eosin stain, ×100). B. high-power view shows the hobnail growth pattern of the epithelial cells which have the nuclear features of papillary thyroid carcinoma in the form of nuclear enlargement, overlapping of the nuclei, irregular nuclear membranes with occasional nuclear grooves. The cells have abundant eosinophilic cytoplasm with oncocyte-like appearance (hematoxylin and eosin stain, ×400).

**Fig. 3:**
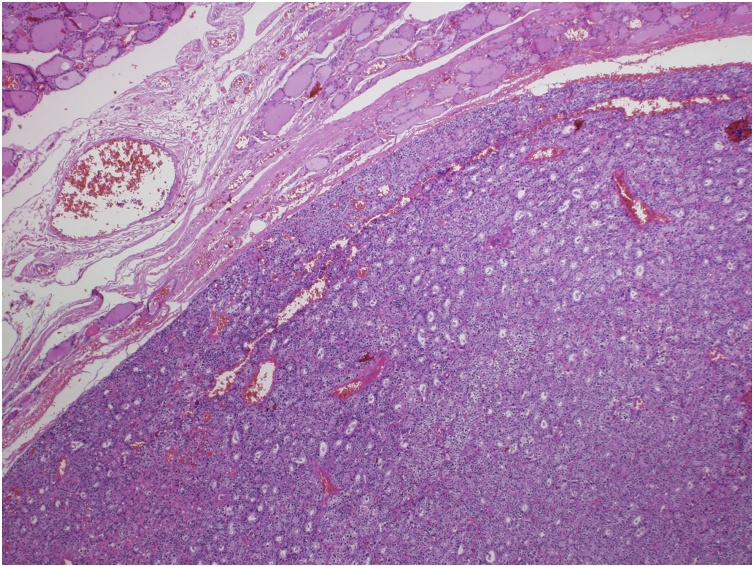
Histological section shows a well-circumscribed parathyroid adenoma with a thin capsule surrounded by thyroid tissue (hematoxylin and eosin stain, ×40).

H&E sections revealed the coexistence of encapsulated non-invasive HPTC measuring 2 cm with all margins uninvolved by carcinoma. No lymphovascular, perineural invasion or extrathyroidal extension were seen. AJCC staging was pT1b (Fig. 2A and B). Also, an ITPA measuring 2 cm completely surrounded by thyroid tissue was identified (Fig. 3).

### 5.2 Immunohistochemical stains

**Fig. 4:**
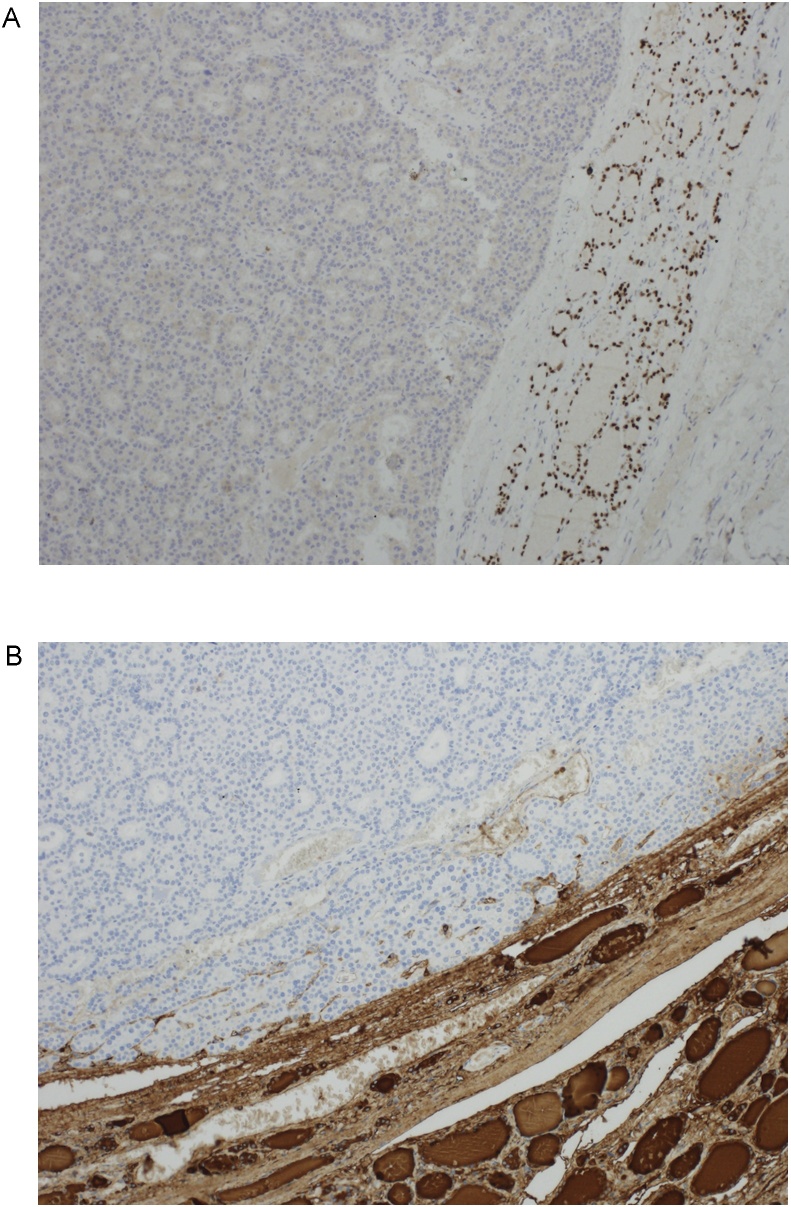
A. TTF1 immunohistochemical stain demonstrates negative staining in the parathyroid adenoma (left) and positive nuclear staining in the adjacent thyroid tissue (right). B. Thyroglobulin immunostain is also negative in the parathyroid adenoma and demonstrates positive staining in the adjacent thyroid tissue.

In the HPTC, the carcinoma demonstrated decreased staining with thyroglobulin and positive staining with HBME-1, galectin-3, and CK19 (controls checked). In the ITPA, TTF-1, thyroglobulin and chromogranin immunostains were performed (controls checked). The parathyroid adenoma was positive for chromogranin but negative for TTF-1 and thyroglobulin, which highlighted only the thyroid tissue (Fig. 4A, B).

**Fig. 5:**
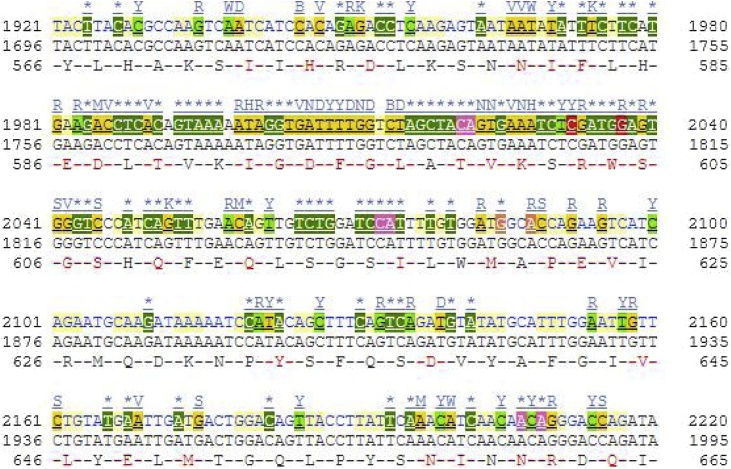
Mutation in *BRAF^V600E^* gene demonstrates result due to a nucleotide change from Thymine (T) to Adenine (A) in codon number 600 in exon 15 of the BRAF gene. This change in nucleotide results in an amino acid change from Valine (V) to Glutamic acid (E). The red arrow shows the position of codon 600 (V) in exon 15.

Sanger sequencing was performed on the amplified target DNA. It revealed a nucleotide change from Thymine (T) to Adenine (A) in codon number 600 in exon 15 of the *BRAF^V600E^* gene. This change in nucleotide results in amino acid change from Valine (V) to Glutamic acid (E). This confirmed the *BRAF^V600E^* mutation in our case (Fig. 5).

## 6 Follow up

The patient had no perioperative complications and was discharged after 2 days. The case was again discussed at the thyroid multi-disciplinary meeting, which decided that the patient’s risk stratification category was low risk (although the case displayed one intermediate risk feature, i.e. aggressive tumor histology hobnail variant) [8]. Hence it was decided that the patient will be under close follow up with ultrasound every 6 months. Neck ultrasound at 6 months revealed that the left lobe was heterogeneous with multiple small hypoechoic nodules, the largest measuring 2.4 × 1.7 mm, and no suspicious lymph nodes. FNAC revealed colloid nodule with cystic degeneration. At 12 months she was completely asymptomatic, with no bone pain, normal calcium and PTH levels, and neck ultrasound and CT scan neck and thorax revealed no metastatic disease or mass, lymphadenopathy or lung metastasis. The patient was satisfied.

## 7 Discussion

HPTC is a recently described aggressive variant of PTC, comprising 0.3%–2.7% of PTCs [2,4,10]. IPTA is documented, but its co-occurrence with PTC is extremely rare [31,32]. HPTC cases have been reported in the literature [4,[9], [10], [11], [12], [13], [14], [15], [16], [17], [18]], and approximately 160 ITPA cases have been reported (2007–2018, Table 1, Table 2). However, only two cases were a simultaneous co-occurrence of PTC and ITPA (Table 3) [31,32]. The coexistence of HPTC and ITPA in the same thyroid lobe has never been reported. This paper reports the first case.

**Table 1:** Review of literature of Intrathyroid Parathyroid Adenoma (2007–2012).

Study*	N	Gender	Age, y	Side	Presentation	Ca (mg/dl)/PTH (pg/mL)	Radiology	FNAC	Treatment	Type of ectopic parathyroid pathology**	Pathology	Postoperative Complications	Outcome
Abboud 2007 Lebanon [42]	6 a	4 F, 2 M	54	2 in superior PT 4 in inferior PT	H, not specified	High Ca and PTH	U/S able to predict 4 cases of ITPA from 6 cases	—	Exploration, 3/6 p subtotal T loboisth mectomy 3/6 p total enucleation of PA	ITPA^b^	3 true type ITPA 3 partial type ITPA	12 to 96 m follow up c	Complete symptoms resolution, N of Ca and PTH
Cheng 2009 USA [36]	1	F	73	L T lobe	Asymptomatic HC and T swelling	11.6/199	99mTc suggest T adenoma, parathyroid lesion could not be ruled out	FNAC: Hurthel cells + lymphocytes consistent with Hashimoto thyroiditis	L HT	ITPA	—	None over 19 m follow up	Remained asymptomatic, N of Ca
Silaghi 2011 Romania [45]	1	F	48	R T lobe	H, HC symptoms, bone pain, weight loss, with 3 cm Ms in inferior R pole of T	13.7/1200	U/S 42 × 27 × 36 mm, inhomogeneous lesion, in R T. 99mTc intense tracer uptake; X-ray osteolytic lesions	—	R HT	ITPA	—	Hungry bone syndrome, tetany requiring lengthy calcium + Vit D Tx & bisphosphonates	Complete symptoms resolution, N of Ca + PTH after 1 year
Herden 2011 Switzerland [41]	4	—	—	—	H, not specified	— d	U/S detected ITPA in one patient; 99mTc done for 3 p, failed to identify any ITPA	—	Started by neck exploration, failed to detect diseased gland, proceeded to HT	ITPA	—	—	—
Goodman 2011 USA [24]	72 e	—	—	90% lower lateral T, 7% posterior of middle part, 3% upper pole	—	—	—	—	—	—	—	—	—
Mazeh 2012 USA [23]	49 f	77% F23%M g	54+/−2 g	48 p clear location identified, 75% inferior, 25% superior g.	40% bone pain28% asymptomatic26% fatigue22% K stones Family and radiation history present in 10% and 4% of p g	11.1/192+/−35^g^	99mTc done for 95% of p and diagnosed ITPA in 70%; U/S done for 35% of p, diagnosed ITPA in 61% g	FNAC done for 5 p. correctly and localized it in 4/5 of them	Bilateral exploration in 28 (53%) p, MIP in 25 (47%) p, and proceeded with TL in 32% of p and enucleation/partial TL in 68% of p	ITPA single (44 p) ITPA Double adenoma (5 p)	—	Complications (12 p); 5 transient Hc; 2 permanent Hc; 2 p hoarseness transient; others mild dysphagia, urine retention g	Complete symptoms resolution, Ca & PTH N
Tanaka 2012 Japan [35]	1	F	58	Middle of R T lobe	Asymptomatic HC	11.4/114	U/S: 13 mm R T lesion suggesting T tumor; 99mTc radiotracer accumulation suggest ITPA	—	R T lobectomy + removal of R upper PT which was normal	ITPA	9 × 6 × 5 mm ITPA	None	Remained asymptomatic, N of Ca & PTH
Heller 2012 USA [40]	50, 13 were true IPTA^h^	—	—	—	H, not specified	—	U/S diagnosed partial ITPA in 25/37 (68%) of p, and complete ITPA in 12/13 (93%) of p	—	Parathyroidectomy (type of procedure not mentioned)	ITPA^h^	—	—	—
Dutta 2013 India [39]	1	F	24	Lower L lobe	H, bone pain, general weakness, O/E 2 × 2 cm palpable Nd in lower L T lobe	12.1/1283	99mTc poor uptake in lower L T; U/S 22 × 18 mm C lesion, suggest simple T C + 6 × 10 mm lesion, posterior to T, suggested PA	FNA-iPTH from adenoma was lower than serum level, FNA-iPTH from suspecting T C shows 3480 pg/mL.	L HT + L inferior parathyroidectomy i	ITPA	C lesion lined by chief cell variant PT cells, surrounded by normal T follicular cells, suggest ITCPA	Tetany	Complete symptoms resolution, Ca & PTH N
Díaz-Expósitoa 2014 Spain [38]	1	F	56	Upper R T lobe	Normocalcemic H, not specified	10.3/105	U/S: single Nd 14 × 11 × 16 mm, mid-upper R T	Colloid Nd	R HT j	ITPA	—	—	N of PTH
Rodrigo 2014 Spain [37]	2	—	—	One in R side (supernumerary), The second not mentioned	H, but not specified	—	—	—	R HT k	ITPA, supernumerary	—	—	Complete symptoms resolution, Ca & PTH N
Mirhosaini 2016 Iran [44]	1	F	29	Inferior pole of L T lobe	Neck swelling suspicious of T Nd	Not done before surgery	U/S: solid HP Ms, 25 × 20 mm in inferior L T	Follicular neoplasia	L HT	ITPA 3 cm	—	Transient Hc	Remained asymptomatic, N of Ca
Shi 2016 China [43]	2	F	59, 45	PTA in R lower T in 1 p, and medial part of L T lobe in other p	First p: neck swelling, Second p: twitching of arms and legs, O/E neck swelling	One p not known Second p: 10.7/182	First p U/S: HP Nd 1.4 × 0.9 cm in R lower T. Second p, U/S: 1.8 × 1.1 cm L T Ms	First p suspected PTC; Second p suspected PA	Surgery l	ITPA	—	—	CA & PTH N
Payá Llorente 2016 Spain [34]	1	F	49	Inferior pole of the R T lobe	Asymptomatic HC on routine labs, Bipolar disorder and on lithium Tx for 20y	11.7/140	U/S: R retro T 1 cm Nd, L T Nd <1 cm. 99mTc: radiotracer accumulation in R lobe (non-ectopic solitary PA?)	—	TT m	ITPA	L T Nd was nodular hyperplasia	None over 6m	No symptoms appeared, N Ca & PTH
Kageyama 2017 Japan [48]	1	F	66	L T lobe	Recurrent pancreatitis (beer drinker), HC; incidental K stones	12.3/253	Contrast CT: 28 mm L T Nd, increased 99mTc uptake	—	L HT	ITPA	—	None over 1 y	No pancreatitis after 1y, Ca & PTH N
Ye 2018 China [25]	12 n	11:4 o	46.2 o	7/12 R T 5/12 L T 6/12 inferior T 4/12 middle T 2/12 superior T	H p	Ca high in 11/12 PTH high in 15/15	U/S detected 9/12 p; 99mTc detected 10/12. Combination of both modalities detected 11/12 p	—	Parathyroidectomy, not specified how	ITPA	—	—	—

*Due to space considerations, only first author is cited; ** after surgery; –: not reported, cannot be inferred; 99mTc (-MIBI): Technetium-99m-methoxyisobutylisonitrile scintigraphy; C: cyst/cystic; Ca: Calcium; F: female; FNA-iPTH: Fine Needle Aspiration intact parathyroid hormone; FNAC: Fine Needle Aspirate Cytology; H: hyperparathyroidism; HC: hypercalcemia; Hc: hypocalcemia; HP: hypoechoic; HT: Hemithyroidectomy; ITCPA: intrathyroid cystic parathyroid adenoma; ITPA: intrathyroid parathyroid adenoma; L: left; M: male; m: months; Ms: mass; mg/dl: milligram/deciliter; N: normalization/normalized; Nd: nodule; O/E: on examination; p: patients; PA: Parathyroid adenoma; pg/mL: picogram/milliliter; PT: parathyroid; PTC: Papillary thyroid carcinoma; K: kidney; MIP: minimally invasive parathyroidectomy; PTH: parathormone; R: right; SPECT: single-photon emission; T: Thyroid; TL: Thyroid lobectomy; TT: total thyroidectomy; Tx: treatment; U/S: ultrasound; y: year.

a 3 were complete type (true type) and 3 were partial type.

b Intrathyroidal parathyroid defined as parathyroid adenoma that is partially/entirely surrounded by thyroid tissue.

c There was transient hypocalcemia in 2 of 178 patients with PA (intrathyroid and extrathyroid). Not clear if any of ITPAs were of these two or otherwise.

d Median values mentioned for all ITPA and extra thyroid PA (115 patients), Ca 11.2 mg/dl, PTH 129 pg/mL.

e These 72 were true ITPA; in another 120 cases, adenoma was partially intrathyroid.

f 101 cases of intrathyroid parathyroid gland disease, include true ITPA, Partial ITPA, and intrathyroid parathyroid hyperplasia, then selected 53 patients with true intrathyroid parathyroid gland, 49 were ITPA and 4 were intrathyroid parathyroid hyperplasia. Analysis was made for 53 cases together.

g These values are applicable to 53 patients (49 patients with ITPA + 4 with intrathyroid parathyroid hyperplasia).

h 144 cases of abnormal intrathyroid parathyroid gland, then selected 53 and categorized to partial ITPA (37 cases), complete ITPA (13 cases), and intrathyroid parathyroid carcinoma (3). The current study deals with only complete ITPA which is well-established term.

i Surgery started by left hemithyroidectomy and there was drop of iPTH just before and after hemithyroidectomy (1054 to 29.4 pg/mL), after confirmation of removal of hyperparathyroidism source, then left inferior parathyroidectomy was performed in same operation.

j started with minimally invasive surgery and intraoperative scintigraphy, parathyroid adenoma discovered (intrathyroid), surgery proceeded to right hemithyroidectomy.

k Patient 1: surgery started by minimally invasive video-assisted parathyroidectomy, failed to find PA. Surgery converted to conventional neck exploration and proceeded with hemithyroidectomy. Patient 2: first operation failed (Minimally Invasive Video-assisted Parathyroidectomy), patient did not improve, imaging localization performed before second operation, then intrathyroid and hemithyroidectomy performed by conventional technique.

l Removed by surgical intervention, but author did not mention type of procedures performed.

m Plan was for right inferior parathyroidectomy but patient asked for total thyroidectomy at same time. Authors did not explain why patient asked for that.

n The article mixes ITPA (12) with 2 cases of intrathyroid parathyroid carcinoma and 1 case of intrathyroid parathyroid hyperplasia and classifies them as true ITPA 12/15 & partial ITPA 3/15.

o ohese values are for 15 patients mentioned in the study, 12 were ITPA, 2 intrathyroid parathyroid carcinomsa, and 1 intrathyroid parathyroid hyperplasia.

p Article mixes clinical manifestation of ITPA (12) with 2 cases of intrathyroid parathyroid carcinoma and 1 case of intrathyroid parathyroid hyperplasia. Among 15 patients, 4 presented with cervical mass, 1 with prolactinoma, 2 with parathyroid mass, 8 presented with osteoporosis, hypercalcemia, bone-arthrosis pain, urinary calculi and thirst complaints.

**Table 2:** Review of literature of coexistence of Papillary thyroid carcinoma and Intrathyroid parathyroid adenoma.

Study*	Gender	Age	Presentation	Pre-operative radiology	Treatment	Pathology	Size (cm)	LN Met	Met
Gürel 2005 Turkey [32]	F	76	Hyperparathyroidism symptoms (lower extremities bone pain)	Radionuclide scan^a^: Hyperactive nodule, right thyroid Hypoactive nodule, left thyroid, suggest ITPA	Left total and right subtotal thyroidectomy, excision of two parathyroid glands	Right side: MPTC Left side: ITPA + 0.1 cm MPTC focus	0.8	—	No
Qasaimeh 2009 Jordan [31]	F	53	Hyperparathyroid symptoms (arthralgia, bone pain)	U/S Neck = 2.1 × 2.3 × 1.3 cm nodule in posterior inferior part of right thyroid lobe, suggest ITPA	Right Hemithyroidectomy + isthemectomy	ITPA PTC	1 × 1 × 0.9 1 × 0.9 × 0.8	Yes	No

–: not reported, cannot be inferred; F: Female; PTC: Papillary thyroid carcinoma; MPTC: Micro Papillary thyroid carcinoma; ITPA: Intrathyroid Parathyroid Adenoma; LN: lymph node; Met: Metastasis.

* Due to space considerations, only first author is cited.

a 99mTc pertechnetate.

**Table 3:** Review of recent literature of Hobnail Variant of Papillary Thyroid Carcinoma*.

Study a	N	F/M ratio	Age, y *M* (range)	Surgical Tx	Radioactive Iodine	Tumor size, mm *M* (range)	Hobnail features *M* (range)	Lymphovascular invasion	Multifocal tumors (%)	Nodal metastasis (%)	AJCC stage	Metastasis	Follow-up *M* (range)	Outcome
Cameselle-Teijeiro 2017 Spain/Portugal [16]	2	1:1	53–62	T thyroidectomy + CLND	Yes	16–65	≥ 50%	Yes	—	50	T3	100%	6–11 y	One died after 6y with metastasis + local recurrence; Second died after 11y with metastasis
Watutantrige-Fernando 2018 Italy [17]	25	3:2	48 (24–73)	T thyroidectomy + CLND	Yes	30 (7–80)	>64% of patients had >30% hobnail feature 36% had 10%–29%	96% of patients	64	68	20%=T1 12%=T2 60%=T3 8%=T4	12%	39 m (13−67 m) (only for 19 patients)	68% had excellent response 32% (6 patients) had disease, (5 P/S, 1 P/B disease)
Song 2018 China [30]	8	—	—	Thyroidectomy b*,*^c^	—	22 (8–46)	—	—	—	—	—	—	—	—
Song 2018 Korea [18] d	2	—	—	T thyroidectomy c	—	—	—	—	—	—	—	—	—	—
Janovitz 2018 USA [49] e	0	—	—	—	—	—	—	—	—	—	—	—	—	—
Nath 2018 USA [50] f	0	—	—	—	—	—	—	—	—	—	—	—	—	—

*Table outlines clinical and pathological characteristics but not molecular profile of the tumor; —: not reported/cannot be inferred; AJCC: American Joint Committee on Cancer 2010 (7th ed.); CLND: Cervical lymph node dissection; F/M Ratio: Female to Male ratio; *M*: Mean; m: months; N: number of cases; P/B: Persistent Biochemical; P/S: persistent structural; PTC: Papillary thyroid Cancer; T: Total; Tx: treatment; Y: years.

a Due to space considerations, only the first author is cited.

b oes not detail whether thyroidectomy was total or otherwise.

c no mention of cervical lymphadenoctomy.

d Authors did not directly report values of individual cases, article examined disease-free survival and dynamic risk stratification of 763 patients with classical PTC (cPTC) and 144 with AV-PTC, including TCV, columnar cell variant and hobnail variants.

e Authors did not directly report values of individual cases, article reviewed aggressive variants of papillary thyroid carcinoma, prognostic significance of vascular invasion in follicular thyroid carcinoma, and Hürthle cell carcinoma.

f Authors did not directly report values of individual cases, article reviewed aggressive variants of papillary thyroid carcinoma including hobnail, tall cell, columnar, and solid variants.

In terms of the presentation, Table 1 shows that ITPA presents predominantly in females with mean age of 52.3 years. Our case was a female (61 years) with classical PHPT presentation (bone pain, polyuria) but no kidney stones. On examination, a right thyroid lobe swelling was discovered, with no compressive symptoms, cervical lymphadenopathy, or distance metastasis. Our patient’s PHPT symptoms agree with Table 1, where more than 50 patients presented with classic hyperparathyroidism symptoms. However, Table 1 shows that some cases were asymptomatic [23,[34], [35], [36]]. There are studies (72 cases) that did not report how patients presented [24]. In the symptomatic group, most studies did not specify the frequency of the symptoms [25,[37], [38], [39], [40], [41], [42]]. An exception is one study where the most frequent symptoms were bone pain (40%), fatigue (26%), and kidney stones (22%) [23]. Our case had right thyroid lobe swelling which supports Table 1, where 6 patients presented with neck swelling [36,39,[43], [44], [45]].

The investigations for a thyroid nodule with hyperparathyroidism are pre, intra and postoperative. Preoperative ITPA diagnosis comprises several options (Table 1). PHPT is present (with high level of serum calcium), however, normocalcemic hyperparathyroidism has been reported [25,38]. Preoperative PTH, although not always reported, is high in all patients when it is performed (Table 1). Ultrasound detected ITPA in 70% of cases [23,25,[40], [41], [42]]. Parathyroid Technetium (99mTc) sestamibi scan localized the ITPA in 70%–83% [23,25]. The combination of ultrasonography and parathyroid sestamibi scan increases the chance of detecting ITPA [25].

The role pre-operative FNA cytological (FNAC) diagnosis in HPTC and ITPA are different. PTC diagnosis is based on The Bethesda System for Reporting Thyroid Cytopathology diagnostic categories [46]. However, FNAC diagnosis of HPTC is challenging, with no consensus on diagnostic criteria, although pre-operative diagnosis of HPTC is possible [2,21]. Others have reported on 10 HPTC cases, all had preoperative FNAC and all had hobnail feature [2]. Asioli et al. undertook FNAC for their 5 cases and all had hobnail feature ranging between 10%–50% [19]. Others performed FNAC (24 patients), where 21 had PTC with Bethesda category IV-V, and another 3 patients were Bethesda III, but they did not report whether the hobnail feature was present [17]. Similarly, others reported 2 cases with pre-operative FNAC diagnostic of PTC Bethesda VI, but again with no mention of the hobnail feature [16]. We are in partial agreement; in our case, preoperative FNAC showed Bethesda III cytology (FLUS). The above findings make it difficult to deduce the role of preoperative FNAC in the diagnosis of HPTC. Future research is required, and a definite HPTC diagnosis is usually confirmed on histopathology after thyroidectomy.

HPTC diagnosis by microscopic examination of the thyroidectomy specimen is also challenging. At low power, the tumor in HPTC usually forms papillary structures that have edematous and/or fibrotic stalks that can be associated with cystic changes [10,15], a feature present in other benign conditions e.g. hyperplastic thyroid nodules. At high power, the classic nuclear features of PTC such as nuclear grooving and pseudoinclusions are less prominent and less common in HPTC [19]. Moreover, cells in HPTC can occasionally have eosinophilic cytoplasm reminiscent of oncocytic cells [15,19]. For these reasons, HPTC can be overlooked or misdiagnosed as hyperplastic thyroid nodule. Since this PTC variant is more aggressive, careful examination of the lesions must be made with attention to the nuclear features of PTC such as nuclear enlargement, nuclear overlapping and nuclear membrane irregularity, in addition to the features characteristic for this variant e.g. discohesiveness of cells, micropapillary pattern and apical location of nuclei within the cell with surface bulge making this “hobnail” appearance.

Likewise, the role of preoperative FNAC diagnosis of ITPA seems not decisive. Whilst preoperative FNAC detected ITPA in 4 out of 5 IPTA patients; FNAC of 2 patients with suspicion that only one of them had ITPA, failed to prove ITPA in the other patient [23,43]. However, Fine Needle Aspiration intact parathyroid hormone (FNA-iPTH) from a suspected ITPA and intrathyroid cystic parathyroid adenoma (ITCPA) showed high iPTH in the ITCPA [39]. The role of FNAC in ITPA is inconclusive. Our preoperative FNAC of the right thyroid nodule revealed FLUS, failing to diagnose the ITPA.

Intraoperatively, rapid PTH monitoring is used to confirm PTA removal [47]. In our case, intraoperative rapid PTH monitoring confirmed ITPA excision, dropping suddenly from 228 to 17 pg/mL (92.5% drop at 5 min) to 13 pg/mL (94.3% drop at 10 min) after ITPA excision. Postoperatively, histopathology provides the definitive diagnosis when this is not accomplished before surgery. In our case, histopathology of right thyroid lobe provided the diagnosis of concurrent HPTC and ITPA.

In terms of the location, most ITPA were located in the lower part of the thyroid gland [23,24]. Table 1 shows 10 left and 13 right IPTAs [25,[34], [35], [36], [37], [38], [39],^44^,^45^,^48^]. Others provided no documentation of the site/side affected [[40], [41], [42]]. We are unable to conclude whether the tumor behavior exhibits preference to a particular side of the thyroid gland. In cases of co-occurrence, our HPTC and ITPA were in the right side, in agreement with reports where PTC and ITPA were both in the right lobe (possibly a coincidence), but in contrast with others where each pathology was in a different lobe (Table 2) [31,32].

The most common mutation occurring in HPTC is *BRAF^V600E^* mutation. This mutation is associated with a higher likelihood of extrathyroidal extension, lymph node metastasis, distant metastasis, recurrence and mortality [2]. Molecular analysis of 10 HPTC cases found *BRAF^V600E^* mutation in 8 cases [2], and in 16 out of 17 cases [3]. Others reported similar results [2,6,19]. *BRAF^V600E^* mutation was also present in our case, supporting the association between *BRAF^V600E^* and HPTC.

In terms of management, the National Comprehensive Cancer Network guidelines indicate total thyroidectomy as primary treatment in PTC patients with any of: radiation history, distant metastasis, bilateral nodularity, extrathyroidal extension, tumor diameter > 4 cm, cervical lymph node metastases, or poorly differentiated features. However, there are no clear current guidelines for the treatment of HPTC [2]. Table 3 depicts that almost all HPTC patients received total thyroidectomy [2,3,6,10,[16], [17], [18], [19], [20]]. However, one patient received hemithyroidectomy [6], and our case is the second patient to receive hemithyroidectomy. This was because our multidisciplinary team concluded that the risk stratification of the patient fitted well in the low-risk category (although it displayed a feature of intermediate risk, aggressive tumor histology, hobnail variant), hence hemithyroidectomy was decided as appropriate.

Table 3 shows that most HPTC patients received variable extents of cervical lymph node dissection ranging from central lymph node dissection to radical neck dissection [2,3,6,10,16,19,20]. Radioactive iodine ablation is also documented, but it is unclear whether such ablation was also undertaken by others [6,[16], [17], [18], [19], [20]]. As we undertook hemithyroidectomy, cervical lymph node dissection or radioactive iodine ablation were not required. There is a gap in the literature regarding the management of HPTC, as many of the above evidence did not clearly document treatment type and details in terms of the type of surgery and extent of cervical lymph node dissection. Future research could benefit from a focus on HPTC treatment and management to provide advice specific for this PTC variant when co-occurring with ITPA.

In terms of prognosis of HPTC, the literature reported high mortality rate, persistence of disease, and high recurrence rate (Table 3). One study reported 2 cases and both died (at 6 and 11 years) [16]. Others reported 10 HPTC patients, where half of the patients died [10]. Likewise, a study of 19 of 25 patients followed for ≈39 months reported that no patients died of the disease, but one third had persistent disease [17]. As for ITPA (Table 1), all patients became asymptomatic after surgery with normalization of Ca and PTH in the follow-up period (range 6–96 months) [23,[34], [35], [36], [37], [38], [39],[42], [43], [44], [45],^48^]. However, others reported that after thyroid surgery for ITPA, few patients had transient hypocalcemia, and very few had permanent/prolonged hypocalcemia needing calcium supplement [23,45]. Our patient had excellent outcomes, no complications, and was asymptomatic at 12 months, with normal calcium and PTH. Her follow-up neck ultrasound of the remaining thyroid lobe found very small non-suspicious nodules. Subsequently, FNAC revealed benign changes of colloid nodule with cystic degeneration in the remaining left lobe, and her CT scan of the neck and thorax revealed no metastatic disease, mass or lymphadenopathy.

## 8 Conclusion

Hobnail variant PTC is extremely rare and its coexistence with intrathyroid PA in the same thyroid lobe has possibly never been reported before. The incidental finding of PTC during parathyroid surgery is rare, and requires a thorough investigation, particularly by imaging in search for any abnormal thyroid findings. The presence of PA should not in any way exclude the diagnosis of thyroid carcinoma. The monitoring of intraoperative rapid iPTH is vital to confirm the complete removal of the parathyroid adenoma. Hemithyroidectomy is appropriate for early stage (pT1b) HPTC coexisting with ITPA in the same thyroid lobe. Describing the cytomorphologic features to distinguish thyroid from parathyroid cells on FNA cytology samples and immunohistochemical stains needs to be considered.

## Declaration of Competing Interest

The authors declare that there is no conflict of interest that could be perceived as prejudicing the impartiality of the research reported.

## Sources of funding

This research did not receive any specific grant from any funding agency in the public, commercial or not-for-profit sector.

## Ethical approval

The Medical Research Centre at Hamad Medical Corporation, Doha, Qatar approved this case series.

## Consent

Written informed consent for publication of their clinical details and/or clinical images was obtained from the patient.

## Author contribution

Omer Al-Yahri: Investigation, Writing - original draft, Writing - review & editing.

Abdelrahman Abdelaal: Investigation, Supervision, Project administration, Writing - review & editing.

Walid El Ansari: Investigation, Supervision, Project administration, Writing - original draft, Writing - review & editing.

Hanan Farghaly: Investigation, Supervision, Writing - review & editing, Validation.

Khaled Murshed: Investigation, Writing - original draft, Writing - review & editing, Validation.

Mahmoud A. Zirie: Investigation, Writing - review & editing.

Mohamed S. Al Hassan: Investigation, Supervision, Project administration, Writing - review & editing.

All authors critically reviewed, revised and contributed to the final article.

## Registration of research studies

N/A.

## Guarantor

Walid El Ansari: welansari9@gmail.com.

## Provenance and peer review

Not commissioned, externally peer-reviewed.
